# Sex-dependent effects of early life sensory overstimulation on later life behavioral function in rats

**DOI:** 10.1038/s41598-024-78928-9

**Published:** 2024-11-12

**Authors:** Abishag Porras, Paolaenid Rodney-Hernández, Jeffy Jackson, Christine H. Nguyen, Millie Rincón-Cortés

**Affiliations:** Department of Neuroscience, School of Behavioral and Brain Sciences, University of Texas at Dallas, Richardson, TX 75080 USA

**Keywords:** Learning and memory, Motivation, Reward, Social behaviour

## Abstract

Children today are immersed in electronic technology shortly after birth as they now begin regularly watching television earlier than they did in the past. Many new programs geared towards infants contain lots of lights, color, and sounds, which may constitute a form of sensory overstimulation (SOS) that leads to cognitive and behavioral changes in children and adolescents. Here, we examined the impact of early life SOS exposure on later life behavioral and cognitive function in rodents by exposing developing male and female rats to excessive audiovisual stimulation from postnatal days (PND) 10–40 and assessing anxiety-like behavior, social motivation, compulsive behavior, and spatial learning/cognition from PND 50–60. To evaluate potential SOS effects on hypothalamic–pituitary–adrenal (HPA)-axis function, levels of the stress hormone corticosterone (CORT) were measured at 3 timepoints (e.g., PND 23, 41, 61) post-SOS exposure. Sensory overstimulated males exhibited reduced anxiety-like and compulsive behavior compared to controls, whereas females exhibited reduced social motivation but enhanced spatial learning/cognition compared to controls. No differences in baseline CORT levels were found at any age tested, suggesting no impact of early life SOS on later life basal HPA-axis function. Our results demonstrate sex-specific effects of early life SOS on distinct behavioral domains in early adult rats.

**Supplementary Information:**

The online version contains supplementary material available at 10.1038/s41598-024-78928-9.

## Introduction

Recent technological advances have contributed to a sharp and significant rise in the usage of screens and other display devices^1–3^. Indeed, digital media can now be consumed via television and computer screens as well as portable phones and tablets, which may lead to excessive media exposure. Excessive media exposure refers to digital media usage that exceeds the exposure guidelines set by the American Academy of Pediatrics, which recommend eliminating digital media exposure (except video-chatting) prior to the age of 2 and 1 h per day for children 2–5 years of age as well as avoiding fast-paced and distracting content^4^. Of note, children today are immersed in electronic technology shortly after birth as they now begin regularly watching television earlier than they did a couple of decades ago, and many new programs are geared towards young infants^5–8^. Since children’s programming contains lots of lights, color, and sounds, this may constitute a form of sensory overstimulation (SOS) during early life, especially in cases of prolonged viewing (i.e., exposure)^9^. Importantly, human studies have found a link between overstimulation in infancy and subsequent deficits in cognition and attention such as increased impulsivity, difficulty in concentration, reduced executive function, as well as delayed socioemotional skills^10–15^. Additionally, there is growing evidence indicating that excessive digital media exposure (i.e., screen time) is linked to detrimental mental health outcomes in children and adolescents, including greater risk for developing psychiatric disorders such as mood, anxiety, and autism spectrum-related disorders^16–21^. Moreover, human studies have shown that greater durations of screen time exposure are correlated with the magnitude of impairments and risk for psychopathology^10,15,22,23^. These findings have raised concerns regarding the impact that excessive digital media exposure and SOS during early life may have on the developing brain as well as later life behavioral function. However, the mechanisms underlying these changes are poorly understood, underscoring an urgent need to understand how excessive digital media exposure, and the accompanying SOS this provides, affects trajectories of behavioral and brain development to influence later life mental health outcomes.

The effects of early life SOS have begun to be examined in rodents using a paradigm that models excessive digital media exposure by continually exposing newborn rodents to audiovisual stimulation^24^. Specifically, mice are exposed to flashing lights that are synchronized with audio from a children’s program for 6 h a day from postnatal days (PND) 10–40^24^. This SOS paradigm is intended to replicate the passive bombardment of audiovisual stimulation that human infants are being exposed to via screen-based devices (e.g., tablets, TV screens). Using this paradigm and variations of this paradigm, multiple groups have shown that overstimulated rats and mice exhibit increased risk-taking behaviors, decreased anxiety-like behavior, hyperactivity, decreased social behavior, learning and memory deficits, and disrupted behavioral responses to cocaine^25–28^. Collectively, these findings indicate that early development is a sensitive period for early life programming effects of SOS on later life behaviors associated with psychopathology, and that the detrimental effects of early life SOS are ubiquitous and span multiple functions including learning, memory, and reward-related processes. Notably, a recent study assessing SOS effects in postpartum female rats found increased licking and nursing of pups, but no effect of anxiety-like behavior or cognition^29^, suggesting a limited impact of SOS in maternal rodents compared to developing rodents and that the previously reported adverse effects of early life SOS are likely not due to deficient caregiving. A limitation of the previous studies is that they were conducted in male animals, so the impact of early life SOS in female animals (along with potential sex differences) remains poorly understood.

**Fig. 1:**
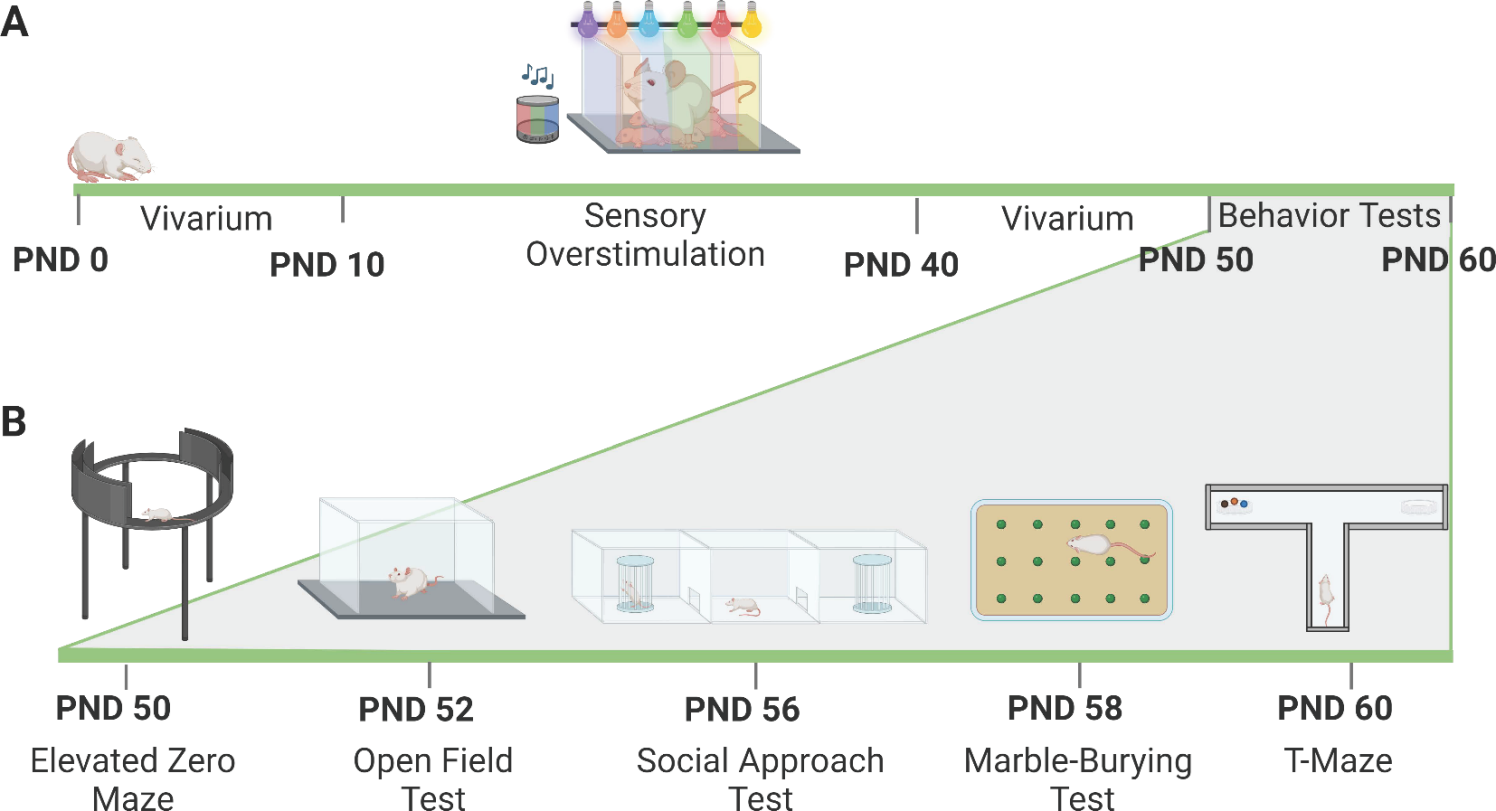
Timeline and Experimental Design. (**A**) Schematic depicting the experimental design and the duration of the SOS paradigm. Briefly, animals were born on PND 0, exposed to control or SOS conditions from PND 10–40 and underwent behavioral testing from PND 50–60. (**B**) Schematic depicting the order for behavioral testing. All animals were tested for behavior in the same order: elevated zero maze, open field, social approach, marble burying and T-maze test. Figure made using Biorender.

This study aims to assess the impact of early life SOS from PND 10–40 on behavioral function in developing rats of both sexes and evaluate whether SOS produces similar effects in males and females. The SOS exposure window (i.e., PND 10–40) was selected based on the initial published studies assessing the impact of early life SOS in rodents^24,26^. After early life SOS, animals of both sexes underwent a behavioral test battery including assays of anxiety-like behavior, social behavior, compulsive behavior, and reward-related spatial learning/cognitive function (see Fig. 1). These tests were selected because previous studies conducted in male rodents (e.g., rats, mice) reported SOS effects in these measures^24,27^, but effects on females remain unknown. In addition, the biological mechanisms underlying SOS-induced changes in behavior are poorly understood. One potential mechanism contributing to the long-term effects of SOS could be dysregulation of the hypothalamic–pituitary–adrenal (HPA) axis. Activation of the HPA-axis results in release of the stress hormone corticosterone (CORT)^30,31^, which is known to modulate anxiety-related behaviors, cognition as well as learning and memory^32–36^. Yet, the impact of early life SOS on HPA-axis function is poorly understood given that only 1 study has measured plasma CORT levels in male SOS mice, and this assessment occurred approximately 10 days after the end of SOS exposure^26^. Here we expanded on this approach by measuring trunk blood serum CORT levels at 3 timepoints throughout development (e.g., weaning, adolescence, adulthood), including during and after SOS exposure. Identifying behaviors disrupted by early life SOS in rodents, including how these may vary by sex, is an important translational goal because it could provide insights as to which brain systems are affected by excessive audiovisual stimulation (and potentially digital media exposure), thereby providing the groundwork for future studies aimed at identifying neurobiological mechanisms driving aberrant behavior following early life SOS.

## Results

### SOS reduces anxiety-like behavior in the elevated zero maze (EZM) in males but not in females

**Fig. 2:**
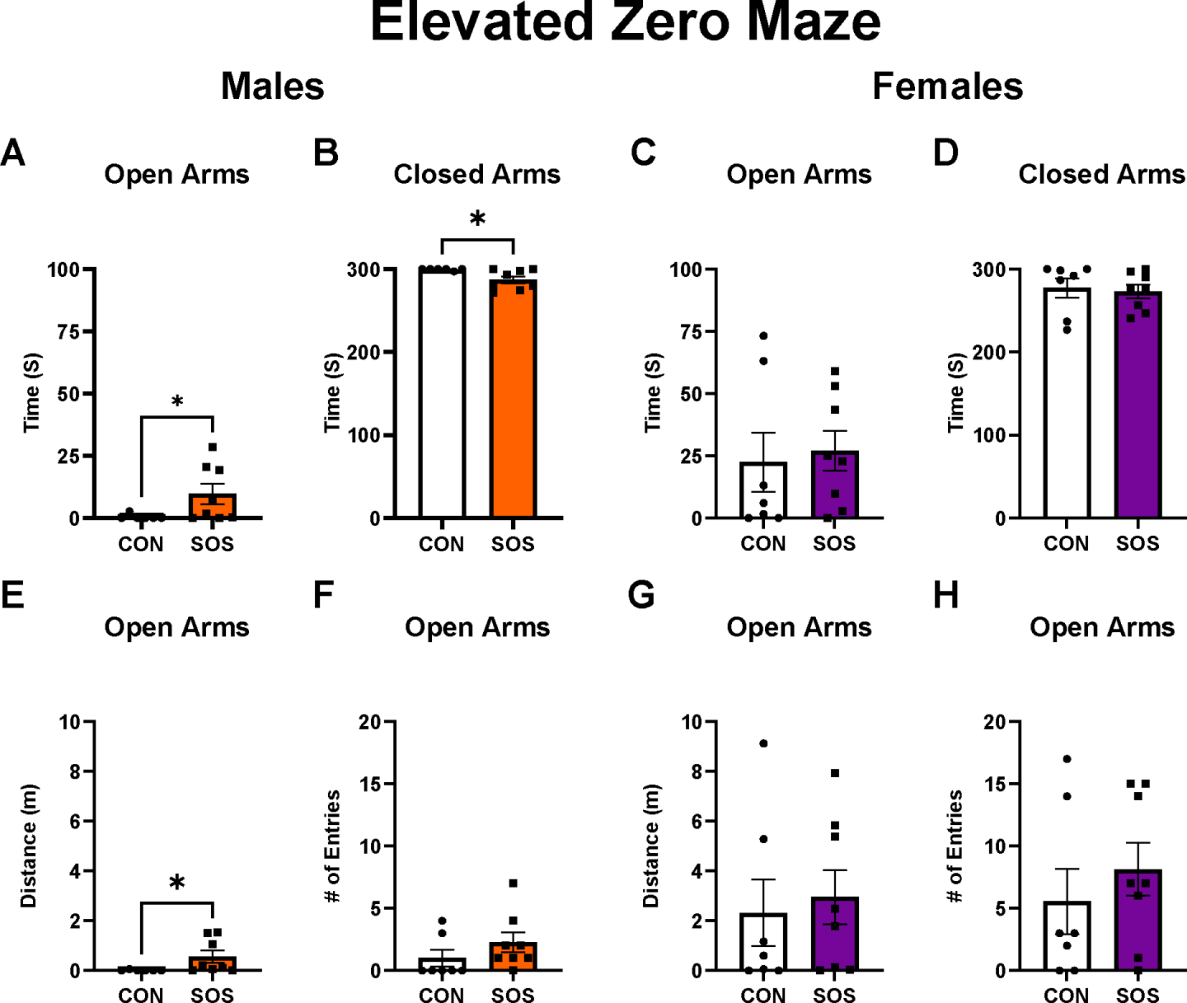
Early life SOS reduces anxiety-like behavior in the EZM in male rats. (**A**) Male rats exposed to early life SOS spent more time in the open arms of the EZM compared to control males (p = 0.04). (**B**) SOS males spent reduced time in the closed arms of the EZM compared to control males (p = 0.01). (**C**) Females exposed to early life SOS exhibited spent comparable open arm time to control females (p = 0.61). (**D**) SOS and control female rats spent similar time in the closed arms (p = 0.61). (**E**) SOS males exhibited an increase in distance travelled in the open arms of the EZM compared to controls (p = 0.03). (**F**) SOS males made similar numbers of entries to the open arms of the EZM compared with controls (p = 0.13). (**G**) Females exposed to early life SOS exhibited similar distance travelled in open arms to control females (p = 0.53). (**H**) SOS and control females exhibited comparable EZM open arm entries (p = 0.41). Error bars represent standard error of the mean (SEM). White bars and circles represent controls, orange bars and squares represent SOS males, purple bars and squares represent SOS females. Each dot or square represents 1 animal (n = 6–8 animals per group). * p < 0.05.

Sensory overstimulated male rats (n = 6–8 per group) exhibited an increase in time spent in the open arms (CON: 0.45 ± 1.10 s, SOS: 9.70 ± 11.43 s; Mann–Whitney; *U* = 9, p = 0.04) and a significant reduction of time spent in the closed arms (CON: 299.6 ± 1.10 s, SOS: 287.1 ± 11.90 s; *U* = 5.50 p = 0.01) of the EZM compared to control males (Fig. 2A, B). Sensory overstimulated female rats (n = 7–8 per group) spent similar time in the open arms (CON: 22.43 ± 31.70 s, SOS: 27.00 ± 22.69 s; *U* = 23, p = 0.61) and closed arms (CON: 277.3 ± 31.54 s, SOS: 273 ± 22.69 s; *U* = 23, p = 0.61) of the EZM compared with control females (Fig. 2C, D). Sensory overstimulated male rats exhibited an increase in distance travelled in the open arms of the EZM (CON: 0.01 ± 0.03 m, SOS: 0.56 ± 0.69 m; *U* = 8, p = 0.03) but similar number of open arm entries (CON: 1.00 ± 1.73, SOS: 2.25 ± 2.25; *U* = 15, p = 0.13) compared to control males (Fig. 2E, F). Sensory overstimulated female rats (n = 7–8 per group) exhibited no differences in distance travelled in the open arms (CON: 2.32 ± 3.54 m, SOS: 2.95 ± 3.07 m; *U* = 22, p = 0.53) or open arm entries in the EZM (CON: 5.57 ± 6.95, SOS: 8.13 ± 6.01; *U* = 20.50, p = 0.41) compared with control females (Fig. 2G, H).

### SOS reduces anxiety-like behavior in the open field test (OFT) in males but not in females

**Fig. 3:**
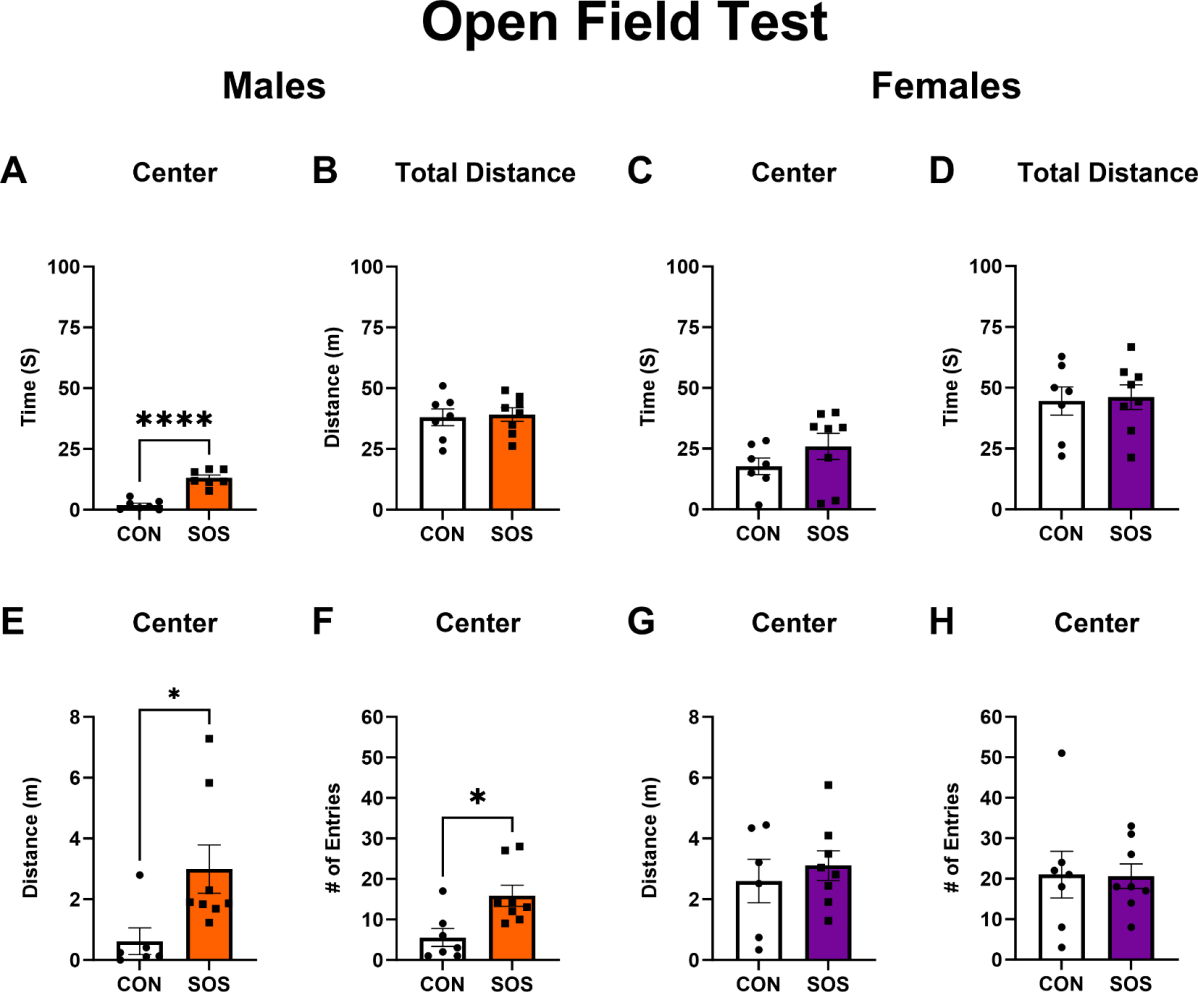
Early life SOS reduces anxiety-like behavior in males in the OFT. (**A**) Males exposed to SOS exhibited increased time in the center of the OFT to controls (p = 0.0001). (**B**) SOS and control males exhibited comparable distance travelled in the OFT (p = 0.80). (**C**) Female rats exposed to early life SOS and controls exhibited comparable time spent in the center of the OFT (p = 0.12). (**D**) SOS female and control rats exhibited comparable distance travelled in the OFT (p = 0.84). (**E**) SOS males travelled greater distances in the center of the OFT compared to controls (p = 0.02). (**F**) SOS males made more entries into the center of the OFT compared to controls (p = 0.01). (**G**) SOS and control female rats exhibited comparable distance travelled in the center of the OFT (p = 0.56). (**H**) SOS and control female rats made similar number of entries into the center of the OFT (p = 0.95). Error bars represent standard error of the mean (SEM). White bars and circles represent controls, orange bars and squares represent SOS males, purple bars and squares represent SOS females. Each dot or square represents 1 animal (n = 6–8 animals per group). * p < 0.05, **** p ≤ 0.0001.

Sensory overstimulated male rats (n = 6–8 per group) spent significantly more time in the center of the OFT (CON: 1.96 ± 1.98 s, SOS: 13.09 ± 3.30 s; t-test: t_12_ = 7.65, p = 0.0001) to controls (Fig. 3A). No between group differences were detected for males (CON: 38.00 ± 9.24 m, SOS: 39.16 ± 7.82 m; t-test: t_13_ = 0.26, p = 0.80) with regards to total distance travelled in the OFT (Fig. 3B). Sensory overstimulated female rats (n = 6–8 per group) spent similar time in the center of the OFT (CON: 17.74 ± 9.02 s, SOS: 25.91 ± 15.24 s; *U* = 14, p = 0.12) compared with controls (Fig. 3C). No between group differences in total distance travelled were detected for female rats (CON: 44.54 ± 15.46 m, SOS: 46.14 ± 14.38 m; t-test: t_13_ = 0.21, p = 0.84) in the OFT (Fig. 3D). Sensory overstimulated male rats exhibited increased distance travelled in the center (CON: 0.61 ± 1.08 m, SOS: 2.99 ± 2.25 m; Mann–Whitney; *U* = 6, p = 0.02) and increased entries into the center of the OFT (CON: 5.57 ± 5.83, SOS: 15.88 ± 7.40; *U* = 6.50, p = 0.01) compared to control males (Fig. 3E, F). Sensory overstimulated and control female rats exhibited comparable distance travelled in the center (CON: 2.60 ± 1.75 m, SOS: 3.11 ± 1.38 m; t-test: t_12_ = 0.61, p = 0.56) and made similar numbers of entries in the center of the OFT (CON: 21.00 ± 15.34, SOS: 20.63 ± 8.62; t-test: t_13_ = 0.06, p = 0.95; Fig. 3G, H).

### SOS exerts a sex-specific reduction of social sniff time (in females) during the 3-chambered social approach test (SAT)

**Fig. 4:**
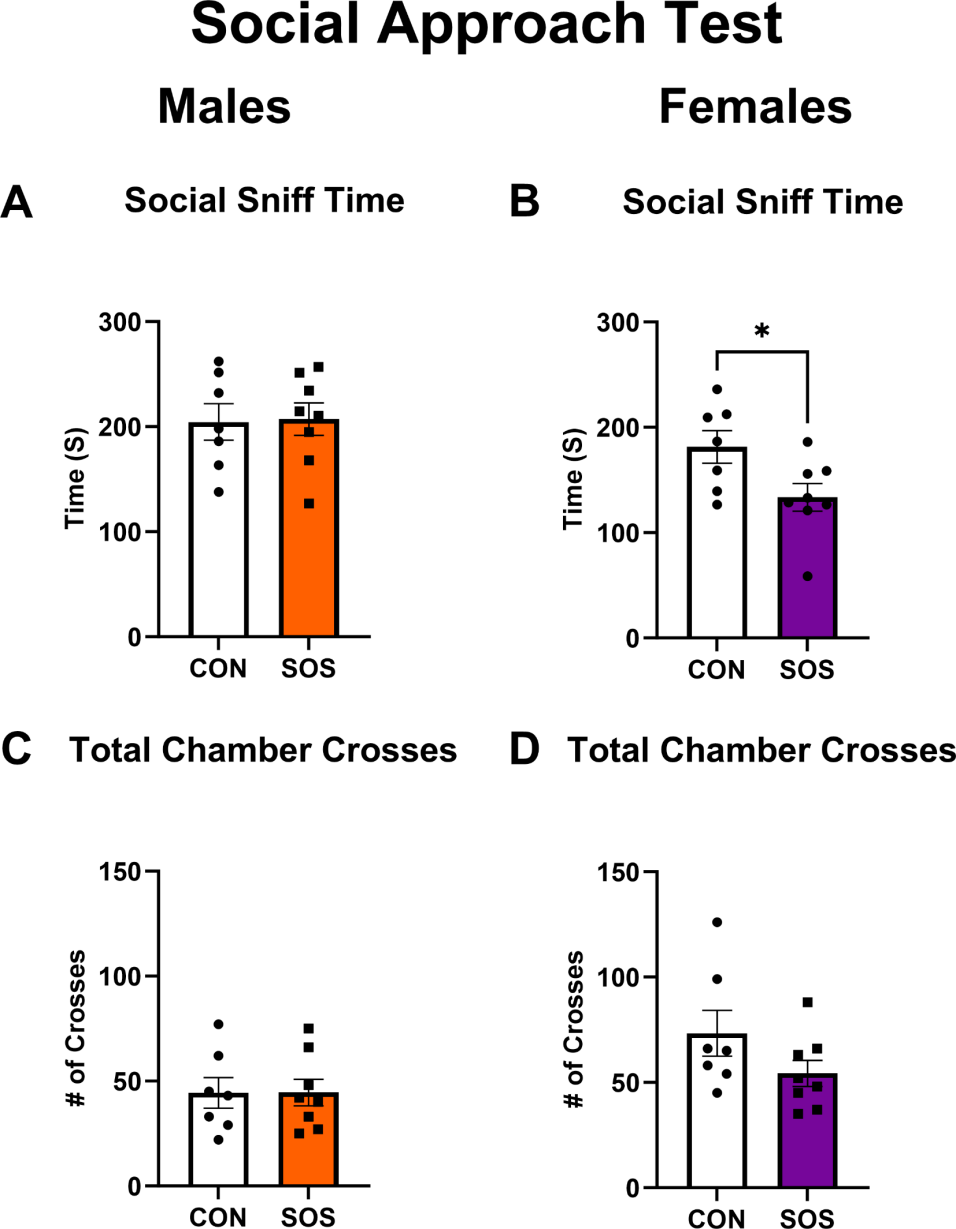
Early life SOS reduces social behavior in female rats during the SAT. Male rats exposed to early life SOS exhibited comparable performance in the SAT, as indexed by: (**A**) similar durations of social sniff time (p = 0.91). (**B**) Female rats exposed to early life SOS exhibited a reduction in social sniff time (p = 0.03) compared to control females. (**C**) Male rats exposed to early life SOS made similar number of total chamber crosses (p = 0.99) compared with control males. (**D**) Female rats exposed to early life SOS exhibited similar number of chamber crosses (p = 0.14) compared with controls. White bars and circles represent controls, orange bars and squares represent SOS males, purple bars and squares represent SOS females. Each dot or square represents 1 animal (n = 7–8 per group). * p < 0.05.

Sensory overstimulated and control male rats (n = 7–8 per group) exhibited comparable social sniff time in the SAT (CON: 204.5 ± 46.18, SOS: 207.1 ± 43.72; t-test: t_13_ = 0.11, p = 0.91; Fig. 4A). However, sensory overstimulated female rats displayed significantly reduced sniff time in the SAT compared to control females (CON: 181.4 ± 40.94, SOS: 133.6 ± 37.27; t-test: t_13_ = 2.37, p = 0.03, Fig. 4B). No between group differences were detected for the number of total chamber crosses in males (CON: 44.43 ± 19.34, SOS: 44.50 ± 17.93; t-test: t_13_ = 0.01, p = 0.99, Fig. 4C) or females (CON: 73.29 ± 28.77, SOS: 54.25 ± 17.53; t-test: t_13_ = 1.57, p = 0.14, Fig. 4D), suggesting that the observed behavioral effects were not due to differences in locomotor activity.

### SOS results in a sex-specific reduction (in males) of repetitive and compulsive behavior in the marble burying test (MBT)

**Fig. 5:**
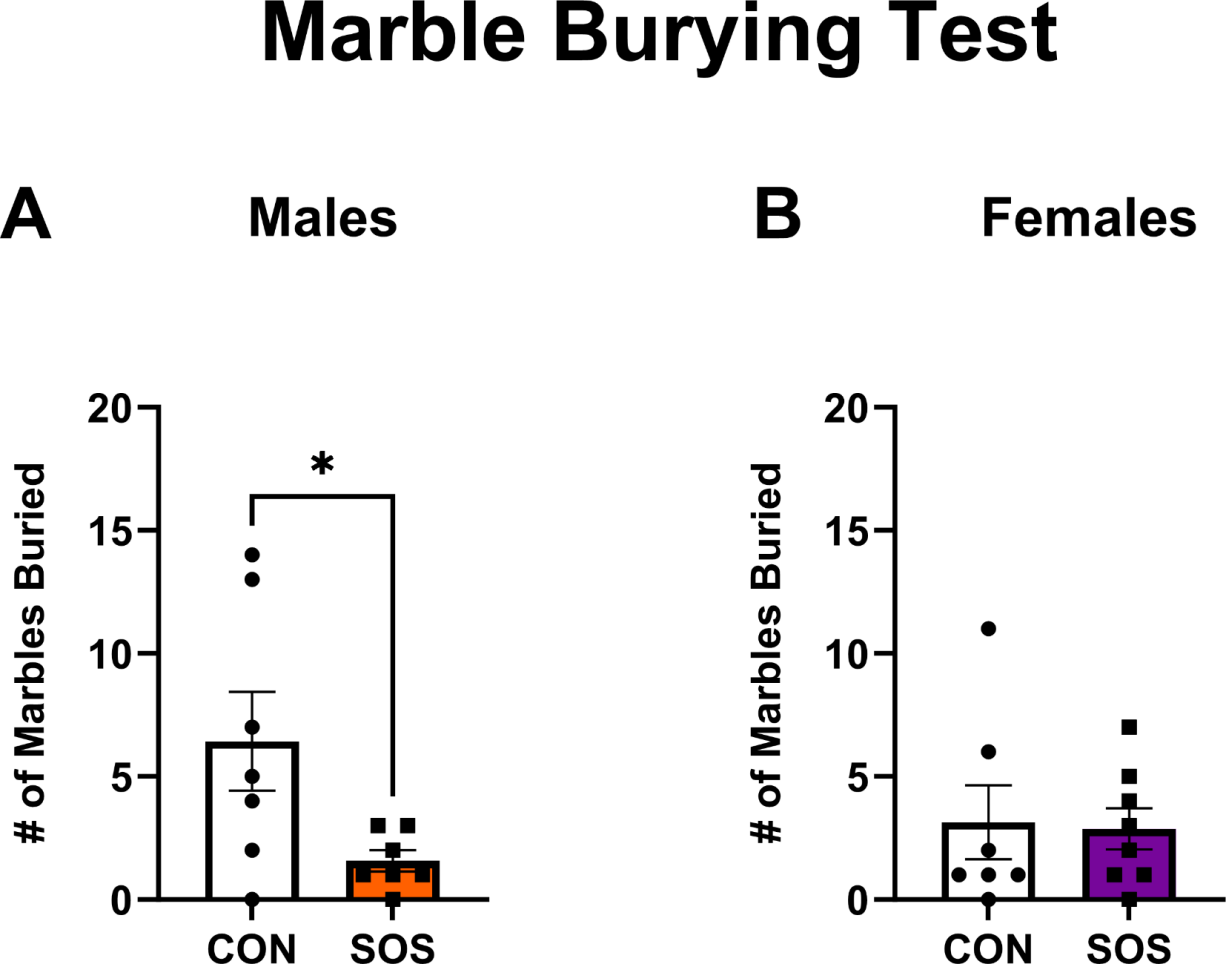
Early life SOS reduces compulsive behavior in the MBT in male rats. (**A**) Male rats exposed to early life SOS buried less marbles compared to controls (p = 0.03). (**B**) Female rats exposed to early life SOS buried similar numbers of marbles compared with controls (p = 0.75). White bars and circles represent controls, orange bars and squares represent SOS males, purple bars and squares represent SOS females. Each dot or square represents 1 animal (n = 7–8 per group). * p < 0.05.

Sensory overstimulated males (n = 7–8 per group) displayed a significant reduction in the number of marbles buried in the MBT (CON: 6.43 ± 5.32, SOS; 1.57 ± 1.13; t-test: t_12_ = 2.36, p = 0.03) compared to control males (Fig. 5A). No differences between sensory overstimulated and control female rats were found for the number of marbles buried in the MBT (CON: 3.14 ± 3.98, SOS; 2.88 ± 2.36; Mann–Whitney;* U* = 25, p = 0.75, Fig. 5B).

### SOS enhances reward-related spatial learning and cognition in the T-Maze in females

**Fig. 6:**
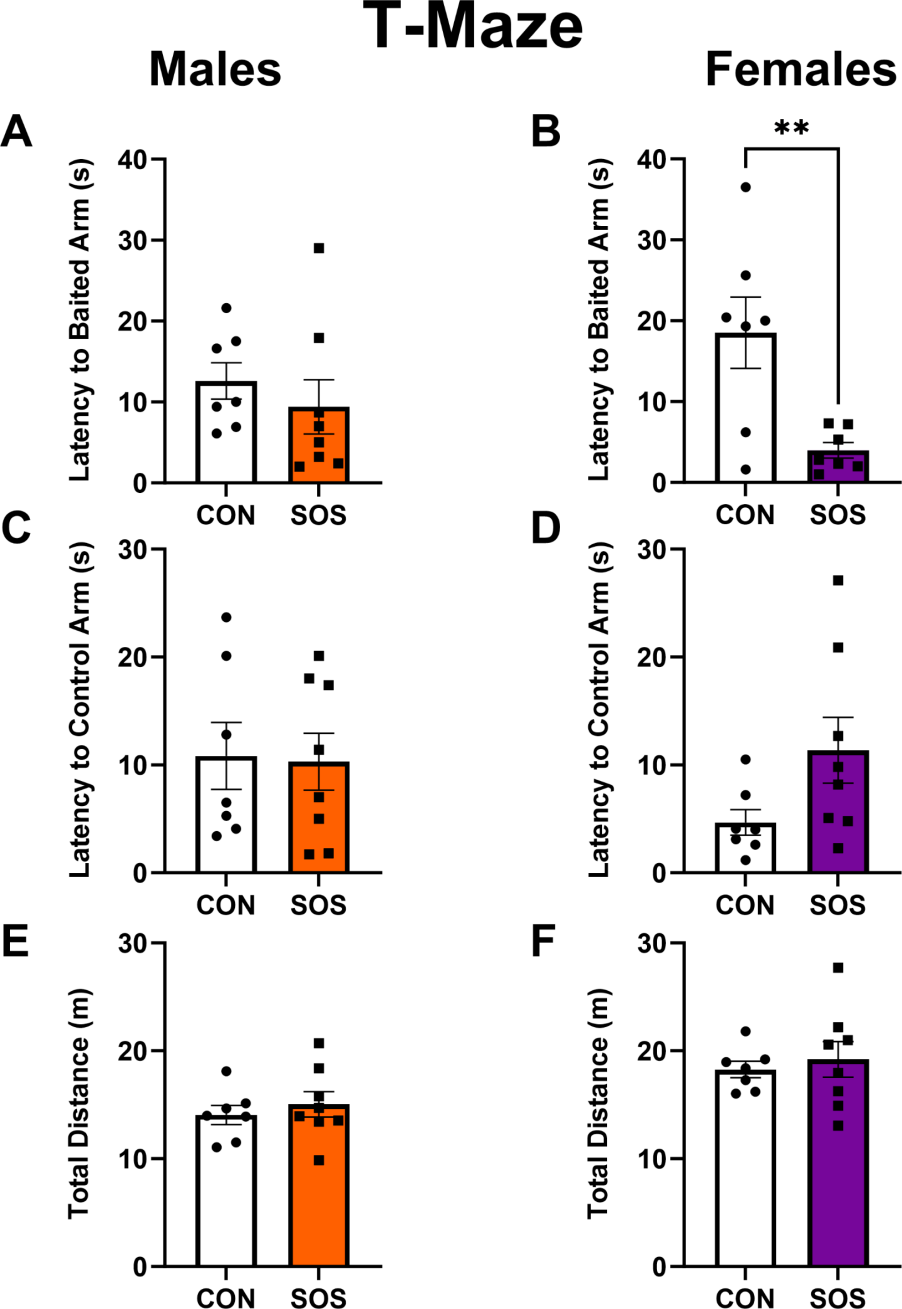
Early life SOS enhances reward-related spatial learning/cognition in female rats. (**A**) Male rats exposed to early life SOS and controls exhibited similar latencies to reach the arm that was previously baited with a reward (p = 0.23). (**B**) Female rats exposed to early life SOS exhibited a reduction in latency to a reach previously reward-baited arm compared to control females (p = 0.007). (**C**) SOS and control males exhibited similar latencies to reach the non-reward baited (i.e., control) arm of the T-maze (p = 0.90). (**D**) SOS and control female rats exhibited no differences in latency to reach the non-baited arm of the T-maze (p = 0.07). (**E**) SOS and control males travelled similar distances in the T-Maze (p = 0.52). (**F**) SOS and control female rats travelled similar distances in the T-Maze (p = 0.63). White bars and circles represent controls, orange bars and squares represent SOS males, purple bars and squares represent SOS females. Each dot or square represents 1 animal (n = 7–8 per group). ** p < 0.01.

Sensory overstimulated male rats (n = 7–8 per group) exhibited similar latency to approach the arm previously baited with a chocolate reward (CON: 12.59 ± 5.96 s, SOS: 9.40 ± 9.44 s; Mann–Whitney; *U* = 17, p = 0.23, Fig. 6A). However, sensory overstimulated female rats displayed a significant reduction in the latency to reach the reward-baited arm (CON: 18.51 ± 11.67 s, SOS: 3.99 ± 2.58 s; t-test: t_12_ = 3.22, p = 0.007 Fig. 6B). No between group differences were detected for males (CON: 10.84 ± 8.22 s, SOS: 10.30 ± 7.45 s; t-test: t_13_ = 0.13, p = 0.90, Fig. 6C) or females (CON: 4.67 ± 3.16 s, SOS: 11.36 ± 8.60 s; t-test: t_13_ = 1.94, p = 0.07, Fig. 6D) in the latency to reach the non-baited (i.e., control) arm of the T-maze compared to controls. No between group differences were detected for males (CON: 14.05 ± 2.36 s, SOS: 15.04 ± 3.32 s; t-test: t_13_ = 0.66, p = 0.52) or females (CON; 18.27 ± 2.0 s, SOS; 19.21 ± 4.67 s; t-test: t_13_ = 0.49, p = 0.63, Fig. 6E, F) with regards to total distance travelled compared to controls in the T-Maze.

### No impact of SOS on CORT levels during weaning, adolescence, or early adulthood

**Fig. 7:**
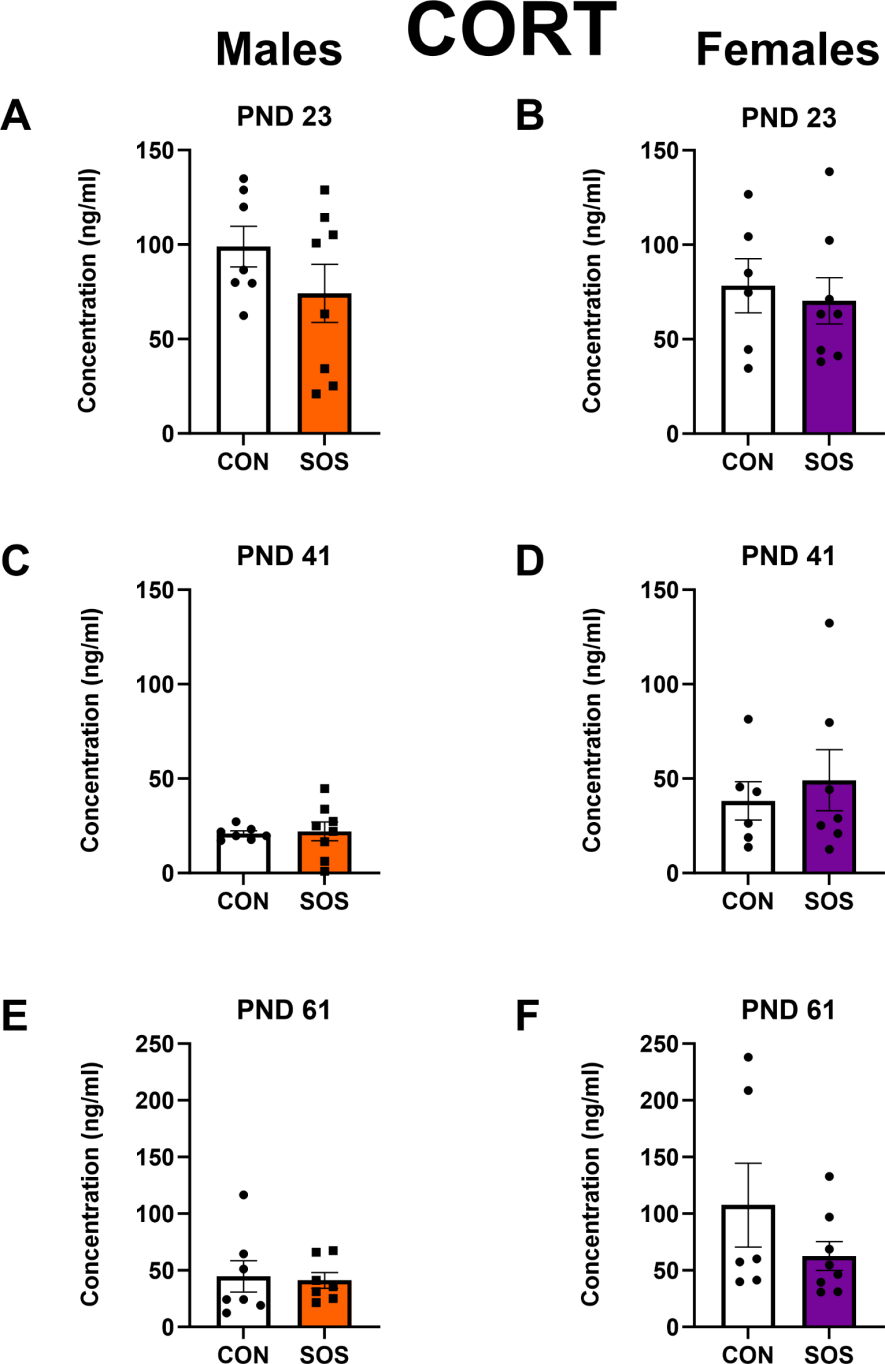
No impact of early life SOS exposure on baseline CORT levels throughout development. (**A**) On PND 23, SOS and control male rats exhibited similar CORT levels (p = 0.22). (**B**) On PND 23, SOS and control female rats exhibited similar CORT levels (p = 0.68). (**C**) On PND 41, SOS and control male rats exhibited similar CORT levels (p = 0.84). (**D**) SOS and control female rats exhibited similar CORT levels (p = 0.59). (**E**) On PND 61, SOS and control male rats exhibited similar CORT levels (p = 0.83). (**F**) On PND 61, SOS and control female rats exhibited similar CORT levels (p = 0.35). Error bars represent standard error of the mean (SEM). White bars and circles represent control groups, orange bars and squares represent SOS males, purple bars and squares represent SOS females. Each dot or square represents 1 animal (n = 6–8 animals per group).

Sensory overstimulated male rats (n = 7–8 per group) showed no differences on serum CORT levels on PND 23 (CON: 98.92 ± 28.46, SOS: 74.16 ± 43.48; t-test: t_13_ = 1.28, p = 0.22, Fig. 7A). Similarly, sensory overstimulated female rats (n = 6–8 per group) showed no differences on serum CORT levels on PND 23 (CON: 78.28 ± 35.00, SOS: 70.29 ± 34.56; t-test: t_12_ = 0.43, p = 0.68, Fig. 7B). No between group differences were detected on PND 41 for males (CON: 20.93 ± 3.51, SOS: 22.05 ± 14.20; t-test: t_13_ = 0.20, p = 0.84, Fig. 7C) or females (CON: 38.15 ± 24.78, SOS: 49.12 ± 42.78; t-test: t_11_ = 0.55, p = 0.59, Fig. 7D) to controls. No between differences were detected for males (CON: 44.59 ± 36.82, SOS: 41.08 ± 18.61; t-test: t_12_ = 0.23, p = 0.83, Fig. 7E) or females on PND 61 (CON: 107.6 ± 90.54, SOS: 62.61 ± 35.88; Mann–Whitney; *U* = 16, p = 0.35, Fig. 7F).

## Discussion

In humans, excessive screen exposure during childhood leads to robust changes in cognitive and behavioral function during adolescence and early adulthood that are linked to increased susceptibility to psychopathology^13,14,18,19,37^. Although many of these effects have been recapitulated in rodent studies^24,26,27,38^, most of this work has been conducted exclusively in male rodents so effects in females (including potential sex differences) remain relatively unexplored. To fill this gap, the current study sought to examine how early life SOS exposure affects behavioral function in male and female rats during later life. Here, we report that early life SOS exposure results in sex-specific effects in distinct behavioral domains in adult rats. Our findings suggest that early life SOS reduces anxiety-like and compulsive behavior in male rats. In female rats, SOS appears to reduce social motivation but enhance reward-related spatial learning/cognition. Collectively, these findings highlight sex differences in the behavioral sequelae induced by excessive audiovisual stimulation during infancy, which we discuss below.

First, our results revealed significant differences between control and SOS male rats with regards to time spent in both the open and closed arms of the EZM (i.e., more time in the open arm, less time in the closed arm) as well as a greater distance travelled in the open arms, suggesting reduced anxiety-like behavior in SOS males. In accordance, we found similar effects in the OFT in which SOS male rats exhibited increased time spent and distance travelled in the center as well as more center entries compared to control males. Thus, SOS induced an anxiolytic effect in males across both tests. These effects were not observed in SOS females, which exhibited similar behavior to control females across all measures of anxiety-like behavior in both tests (i.e., EZM, OFT). We found no differences in total distance travelled in the OFT, suggesting that reduced anxiety-like behavior in SOS male rats was not due to differences in general locomotor activity (i.e., no hyperactivity/hypoactivity compared to control males). Importantly, our results in male SOS rats replicate the direct measures utilized in the original study conducted in mice^24^ such as increased distance travelled in the open arm of the EZM, distance travelled in the center, and number of entries into the center of the OFT. These results validate findings from the original study, which proposed that early life SOS leads to reduced anxiety-like behavior during later life in male mice^24^ and extend these findings by highlighting sex differences in SOS effects on later life anxiety-like behavior. Collectively, these findings point to sex-specific changes in anxiety-like behavior following early life SOS, suggesting an anxiolytic effect in male but not female rats.

Research in humans suggests that excessive screen exposure could deter humans from engaging in social behavior and decrease social coping skills^39–42^. Examining the extent to which SOS effects in rodents parallel clinical literature is imperative for understanding environmental effects on social behavior. A prior study showed that rats exposed to excessive early life audiovisual stimulation exhibited reduced sniff time to a stranger rat compared to a novel empty cage, suggesting deficient social behavior^27^. After evaluating the impact of early life SOS on adult social behavior, we found significant differences in social sniff time in females but not males. Specifically, SOS females exhibited reduced social sniff time compared to control females. The lack of effect seen in SOS males in the current study may be due to several factors, including strain differences (i.e., Sprague Dawley vs Wistar), protocol variations (i.e., distinct duration of SOS exposure) and different comparison groups. Moreover, our protocol consisted of exposing the test animal to a novel and younger same-sex rat that had previously been habituated to the enclosure (to avoid potential stress and aggression effects) and a plastic rat toy, leaving no enclosure completely empty. Furthermore, our test was conducted in slightly older animals of both sexes (PND 56) than the male animals used by Mansouri and colleagues (PND 42). Finally, the Mansouri study employed different groups and statistical analyses given that they were also examining the impact of maternal separation (MS) and of combined MS and SOS on a variety of other behavioral tests.

In humans, increased screen time has been associated with higher odds for developing obsessive compulsive disorder, which is a mental health disorder characterized by a compulsive desire to repeat a behavior^43^. In rats, compulsive behavior is usually assessed through marble burying, in which an increase in the number of marbles buried serves as an index of greater compulsive-like behavior^44,45^. Although a previous study showed that SOS male Wistar rats did not exhibit compulsive behavior, as indexed by comparable levels of marble-burying compared to control males^27^, here we report a sex-specific reduction of marble burying in SOS males compared to their control counterparts but not in SOS females. The present findings suggest that compulsive-like behavior is reduced in male SOS rats and may point to resilience towards developing this phenotype in Sprague Dawley rats. Interestingly, although Mansouri and colleagues (2022) found that excessive early life audiovisual stimulation alone did not increase marble burying, the combination of excessive early life audiovisual stimulation and MS significantly increased marble burying. This suggests that multiple developmental “hits” may be necessary to produce increased compulsive-like behavior in this test. Further, although we used the marble burying test to assess compulsive behaviors, previous studies^46,47^ have attributed the burying behavior to anxiety-like or stress responses. Within this context, we could interpret our results as suggesting a sex-specific anxiolytic effect in SOS male rats, as indexed by a reduction in marble burying, but not SOS female rats. Indeed, these findings correspond with our results seen in the EZM and OFT for SOS male rats, reinforcing the idea that a sex-specific anxiolytic effect exists in male rats following early life SOS.

Various studies in rodents and humans have demonstrated sex differences in learning and cognitive strategies, which can influence decision-making processes^48,49^. Task specific disparities also exist with male rodents outperforming females in certain spatial tasks like spatial navigation on the Ziggurat task, but not in the Morris water task^50^. Here, we used the T-maze to evaluate reward-related spatial learning/cognition and found a sex-specific effect in SOS females. SOS female rats exhibited a reduced latency to approach the arm of the T-Maze that was previously baited with a chocolate reward compared to controls. This effect was specific to the baited arm as SOS and control females displayed similar latencies to reach the control arm (not previously paired with reward). However, SOS males did not show any significant differences in latency to reach the previously reward-baited arm or the non-baited arm compared to controls. Taken together, these data may suggest enhanced reward-related spatial learning and cognition in SOS females. These findings are consistent with literature indicating that female rodents exhibit enhanced reward-seeking behaviors and greater motivation to obtain sugar-containing palatable foods, whereas male rodents exhibit distinct response strategies^51–53^.

Finally, understanding how the HPA-axis is responding to early life SOS could provide insights as to whether excessive audiovisual stimulation during infancy may constitute a form of early life adversity. For example, early life adversity typically dysregulates HPA-axis function and exerts long-term effects on brain areas/systems regulating a multitude of behavioral processes to influence risk for psychopathology^54,55^. Yet, the impact of early life SOS on HPA-axis function is poorly understood given that only one previous study measured plasma CORT levels in male SOS mice approximately 10 days after SOS exposure, and found no differences in CORT levels compared to control (i.e., unstimulated) mice^26^. Some limitations of this study included the lack of female animals and that CORT levels were assessed after the end of the SOS exposure period but not during the SOS exposure. To build upon this prior study and address these limitations, our study assessed the effects of early life SOS exposure on HPA-axis function by measuring CORT levels in rats of both sexes across 3 developmental timepoints (e.g., weaning, adolescence, adulthood). We report no difference in baseline serum CORT levels at any of these timepoints, suggesting that early life SOS exposure does not act as a chronic environmental stressor. Thus, the sex-specific behavioral effects observed in SOS rats do not appear to be mediated by alterations in baseline CORT levels. These findings are in agreement with prior studies showing no differences in baseline CORT levels following SOS exposure in male mice or rat dams^26,29^. However, a limitation of this and previous studies is that none of them have measured CORT levels in response to a stressor, so it is possible that SOS influences HPA-axis function in this context. This possibility should be explored in future studies.

In sum, we conducted the first study assessing long-term effects of early life SOS in both male and female rats. We report sex-dependent behavioral effects of early life SOS during later life in which males exhibit reduced anxiety-like and compulsive behavior and females exhibit reduced social motivation but increased reward-related spatial learning/cognition. It is worth mentioning that we do not think these effects are due to aberrant (i.e., deficient) maternal behavior in SOS dams. In fact, a recently published study showed that SOS dams exhibit an enhancement in a subset of maternal behaviors compared to control dams, as they spent higher percentages of time engaged in positive caregiving behaviors that are beneficial to pups, such as nursing and licking of pups^29^. This would suggest that there are other mechanisms (besides adverse maternal behavior) driving the long-term effects of early life SOS. Elucidating the exact mechanism by which early life SOS is associated with these sex-specific behavioral outcomes needs to be better understood to shed light into how SOS-induced functional and physiological changes may render humans vulnerable to later life psychopathology. Future directions for this study include understanding how early life SOS changes the brain to promote resilience to anxiety-like behavior in males or buffer against the social dysfunction observed in females. For example, future studies should examine changes within the social brain in SOS females displaying deficient social behavior. Since social behavior deficits can often manifest as a lack of interest in social interaction or avoidance of a social situation, this can be indicative of a reduction in motivation resulting from mesolimbic dopamine (DA) system dysregulation^56–58^. Thus, future studies aiming to probe neural correlates of SOS-induced social dysfunction, may wish to evaluate changes in mesolimbic DA system function, which is known to mediate various aspects of social behaviors^59,60^. Finally, this study serves to establish a rat paradigm for modeling passive screen exposure in rodents to inform a rising clinical issue (i.e., long-term effects of excessive audiovisual stimulation during early life on brain and behavior) that is yet to be fully understood.

## Methods and materials

### Animals

Primiparous timed-pregnant adult Sprague Dawley rat dams were obtained from Inotiv on gestational day (GD) 14, housed individually and maintained in a quiet, temperature-controlled room on a 12-h reverse light/dark cycle (6 AM lights off /6 PM lights on) with unlimited access to food and water. Parturition was verified daily starting on GD 20–23. The day of birth was designated as PND 0. Litters were culled to 9–10 pups with an equal sex distribution (i.e., 5 males and 5 females) whenever possible on PND 2, at which point they were randomly assigned to experimental conditions (control or overstimulation) using simple randomization (i.e., coin flip). On PND 23 litters were weaned into same-sex and condition-matched cages. All animals remained in their assigned experimental conditions until PND 40. A total number of 149 rats were used in these experiments (please see Supplementary Table 1 for additional information). Multiple cohorts (n = 4 cohorts, 4 per group, 2 per sex) of both male and female animals were utilized for all behavioral tests in this study. All experiments were carried out according to NIH guidelines and were approved by the University of Texas at Dallas Institutional Animal Care and Use Committee. Experimental procedures and results are reported in accordance with the ARRIVE guidelines^61^.

### Sensory overstimulation paradigm (SOS)

One of the mechanisms by which excessive early life digital media exposure may exert its deleterious effects is through the flashing lights, scene changes, quick edits, and auditory cuts characteristic of children’s programming, which may constitute a form of SOS for the developing brain^9^. The SOS paradigm was originally designed to mimic the audiovisual stimulation human children and mothers experience when watching television on a screen at home and consists of exposing the litter (dam and pups) to flashing multicolored LED lights and an audio recording of a children’s program during a prolonged period of time (6 h per day) from PND 10–40^24,38^. Thus, this paradigm is focused on the SOS aspects (i.e., lights, sound) that passive screen viewing provides, rather than screen viewing itself.

Litters remained in standard housing conditions until the start of the SOS procedure on PND 10. Litters assigned to control conditions remained in the vivarium, whereas litters assigned to the SOS condition were transported to a designated SOS room. Control and SOS animals were housed in ventilated cages placed on racks within the same room. Aside from animal husbandry performed by our laboratory staff, animals were left undisturbed. SOS animals were removed from the same racks the controls were placed on before being transported to the SOS room to undergo a 6-h SOS exposure period. SOS animals were returned to the same room as the control animals immediately after the end of the SOS period each day. Once in the SOS room, animals were placed on a rack consisting of 4 shelves, 2 of which had a LED strobing light speaker placed on them (Megatek-Dual portable wireless Bluetooth speakers). The audio used consisted of a layered track of children’s programming (i.e., SpongeBob SquarePants episodes) and the sound level was kept below 70db, which is lower than the levels used in audiogenic stress models (i.e., 90-105db)^62,63^. Gradient multicolor (LED) lights were wrapped around the entire rack and kept on the flashing mode feature throughout the entire exposure period. The entire litter (dams and pups) were exposed to the sound and flashing lights for 6 h a day 7 days a week, typically between 9:00AM-3:00PM, as previously described^29^. On PND 23, pups were weaned into same-sex groups of 2 or 3 and continued to receive the SOS until PND 40. PND 10–40 was chosen based on the initial published studies assessing the impact of early life SOS in rodents^24,26^. After pups were weaned, the dams did not receive additional SOS exposure. Control litters remained in the vivarium from PND 10–40. A behavioral test battery was used to evaluate anxiety-like behavior, social motivation, compulsive behavior, and spatial learning/cognition from PND 50–60 in both male and female rats (see Fig. 1).

### Behavioral testing

Control and SOS rats of both sexes underwent a behavioral test battery from PND 50–60, with most tests occurring every other day in sequential order within the same behavior room (see Fig. 1). Testing occurred between 9:00 AM and 4:00 PM during the animal’s dark cycle under red light. Animals received a 1-h habituation to the test room prior to the start of each test. All rats underwent behavioral testing in the following order: elevated zero maze (EZM), open field test (OFT), social approach test (SAT), marble burying test (MBT), and T-maze. Sex and condition were counterbalanced whenever possible across the behavioral tests. Animals were tested in the same time period throughout each cohort to control for time-of-day effects on behavioral testing. Behavioral tests were videotaped and scored via Any-Maze (EZM, OFT, SAT, and T-Maze) or an experimenter blinded to the animal’s condition (marble burying). All behavioral apparatus (arenas, mazes) were cleaned with 70% ethanol in between animals.

#### Elevated zero maze

Rats were tested for anxiety-like behavior in the EZM on PND 50. The EZM consisted of an "O" shaped platform (10 cm × 100 cm × 61 cm) raised above the ground and divided into two sections: one with walls 30 cm high surrounding the edges (closed sections) and two sections with no surrounding walls (open sections). On test day, each rat was placed into a closed arm and allowed to explore the maze for a 5-min period. Behavior was recorded with an overhead video camera and stored on a desktop computer and analyzed using automated behavioral tracking (i.e., Any-Maze) software. In this test, increased time spent in the open arm and/or reductions in time spent in the closed arm are interpreted as decreased anxiety-like behavior^64,65^.

#### Open field test

Animals were tested for anxiety-like behavior in the OFT, which consisted of an open top acrylic apparatus (60 cm x 60 cm x 40 cm), on PND 52. On test day, animals were placed into the corner of the open field apparatus and allowed to explore the arena for 10 min. Animals were placed in the corner instead of the center to ensure that the rat was voluntarily choosing to spend time in the center^66^. Behavior was recorded with an overhead video camera, stored on a desktop computer, and analyzed using Any-Maze tracking software to determine time spent in the center of the open field, distance travelled in the center, number of entries into the center, and total distance travelled. In this test, greater measures are interpreted as a decrease in anxiety-like behavior whereas reductions in center time are thought to reflect increased anxiety-like behavior^67–69^.

#### Social approach test

Animals were tested for social motivation in the 3-chambered SAT on PND 56. This apparatus consisted of two larger side chambers (40.5 cm by 80 cm), each of which contained cylindrical enclosures (15 cm in diameter, 30 cm high), and a smaller center chamber. The test rat was placed on the center chamber and the time exploring the side chambers as well as the cylindrical enclosures (one containing a social stimulus animal, the other one containing an inanimate object) was recorded for 10 min^70–72^. Social stimulus animals consisted of younger and novel same-sex rats that were habituated to the cylindrical enclosure inside the chamber for 10 min on the day prior to conducting the SAT. On test day, both experimental and social stimulus animals were transported to the room for a 1-h acclimation period to the test room. Following acclimation to the room, experimental animals were also habituated to the 3 chambers for 5 min by being placed in the center chamber and allowed to run freely and explore all three chambers. After the 5-min habituation to the apparatus, the test rat was removed and a social stimulus animal was placed inside one of the cylindrical enclosures, and a plastic toy rat was placed inside the other enclosure to serve as a novel object control. The test rat was then placed in the center chamber and allowed to freely explore for 10 min. All trials were recorded with an overhead video camera, saved on a desktop computer, and scored using Any-Maze. Behavioral measurements scored included: the amount of time that was spent sniffing the social stimulus animal, which refers to the amount of time the animal spends in close proximity to the social stimulus cage (or enclosure) with its snout oriented towards the cage and serves as an index of social motivation^71–73^; and the number of times the animal crossed into each of the 3 chambers, which serves as a general index of locomotor activity^74,75^.

#### Marble burying test

Animals were tested for compulsive behavior using the MBT on PND 58. In this test, animals were presented with a set of 20 marbles in their home cage, which was given 1,000 ml of additional bedding 24 h prior to testing to allow sufficient room for burying. Each rat was tested individually during a 30-min test in which they were evaluated based on their tendency to bury the marbles. Immediately after the test period, the rat was removed from the home cage and a photograph was taken of the home cage and saved on a desktop computer to score the number of marbles buried. A marble was scored as buried if 2/3 of its volume was covered by bedding^76^. Burying or digging of marbles has been utilized as a measure of compulsive behavior in rodents^77–80^.

#### T-maze

Animals were tested for reward-related spatial learning/cognition using the T-Maze on PND 60. In this test, 2 ramekins were placed inside the maze. Each arm (50 cm length × 10 cm width) contained a ramekin: one that was baited with 3 M&M pieces, while the other arm contained an empty ramekin. The location of the M&M bait was counterbalanced to control for any side preferences. The animals were placed at the base of the maze and allowed to freely explore the T-maze containing baited and non-baited arms for 10 min. This allowed the animal to associate an arm with a food reward and distinguish between reward and non-reward related arms, which is thought to reflect spatial learning/cognition^51,81^. After the 10-min, rats were given a 30-min inter-trial interval (ITI) before being tested. During the 5-min test, the rat was placed at the base of the maze in which both arms of the maze had empty ramekins that were wiped down with 70% ethanol to ensure no carryover odors from previous contents. All trials were recorded with an overhead video camera, saved on a desktop computer, and scored with Any-Maze software. The latency to reach both right and left arms of the T-maze, and the total distance travelled during the test period was scored^82^. In this test, a shorter latency to reach an arm indicates shorter recall and better spatial learning/cognition as opposed to a longer latency time, which may serve as an indication of impairments in reward-related spatial learning/cognition^83^.

#### Any-maze tracking

Following behavioral testing, recorded videos were analyzed using Any-Maze. To do this, experimenters opened the recorded videos on the behavioral tracking program, recorded animal movement in outlined zones of the apparatus, and defined investigation zones for each behavioral test (i.e., EZM, OFT, SAT, T-Maze). The center of the animal was used to track movement into zones in the EZM (i.e., closed vs open arms) and OFT (i.e., periphery vs center) as well as throughout the entire apparatus. Snout orientation and center of body was used when scoring animal interaction with an object or social stimuli animal (e.g., SAT, T-Maze). Test specific measures were defined in each protocol prior to running of recorded behavioral tests.

### Trunk blood collection and serum extraction

Animals used for assessment of CORT levels were rapidly decapitated at 3 different time points (e.g., PND 23, PND 41, PND 61) from 11:30am-2:30 pm. No anesthetic agents were used in these animals because anesthetics are known to influence (introduce a confound into) stress hormone (i.e. CORT) levels, which we were measuring^84,85^. Trunk blood was collected within 2 min of cage disturbance to minimize stress artifacts on CORT measurements^86^. Blood was collected into a 2 ml Eppendorf tube and placed on ice immediately. Following the blood collection period, the samples were allowed to sit at room temperature and coagulate for 1 h. Samples were then centrifuged in an Eppendorf 5425R centrifuge with a rotor FA-24×2 (radius of 6.5 cm) for 15 min at 4 °C, 3200 rpm or 744.14 rcf. The resulting supernatant (serum) was aliquoted into 0.5 ml Eppendorf tubes and stored at -80 °C until an ELISA assay was performed as previously described^29^.

### CORT measurements

Serum concentrations of CORT were determined using a CORT ELISA kit according to the manufacturer’s instructions (Enzo Life Sciences Co., Farmingdale, New York). The sensitivity of the assay was 0.03 ng/ml for CORT. CORT sample optical densities were assessed using a microplate reader (accuSkan FC, ThermoScientific). Coefficient of variance percentages (CV%s) were calculated for each sample replicate. Only CV%s < 10% were used. Sample concentrations were then interpolated from the optical densities using a four-parameter logistic curve fit on GraphPad Prism 9.5.1. Standard dilution accuracy was validated by reverse interpolating standard concentrations and comparing these to the expected standard concentrations.

### Statistical analysis

Sample sizes were calculated a priori following collection of preliminary data using G*Power^87,88^, in which determined that we needed an n = 6 rats per group to achieve 80% power. Behavioral data with normal distributions were analyzed using unpaired t-tests; data sets diverting from the normal distributions were analyzed using Mann–Whitney *U* tests. Statistics were calculated using GraphPad Prism 9.5.1 and differences were considered significant at p < 0.05. Statistical outliers were identified using QuickCalc Grubbs test (GraphPad) and excluded from analysis.

## Electronic supplementary material

Below is the link to the electronic supplementary material.

Supplementary Material 1

## Data Availability

Data generated and analyzed during this study are included in this published article.
