# Supplementary material for: Sex-dependent effects of early life sensory overstimulation on later life behavioral function in rats
Source: Sci Rep. 2024 Nov 12;14:27650. doi: 10.1038/s41598-024-78928-9 (PMC11557974; doi:10.1038/s41598-024-78928-9)
Supplement: Supplementary file 1 — Supplementary Material 1 [file 41598_2024_78928_MOESM1_ESM.docx]

| **Condition** | **Dams** | **Males** | **Females** | **Behavior Only-Males** | **Behavior Only-Females** |
| --- | --- | --- | --- | --- | --- |
| SOS | N=8 | N=37 | N=43 | N=8 | N=8 |
| Control | N=7 | N=35 | N=34 | N=8 | N=8 |

**Supplementary Table 1 Distribution of animals used for the current study.** N refers to the number of animals used. N numbers under male and female columns indicate number of animals used for trunk blood collection for corticosterone (CORT) analysis.
